# Notes of the Modern Treatment of Fractures

**Published:** 1900-07

**Authors:** 


Notes of the Modern Treatment of Fractures.—By John B. Roberts, A. M., M. D., Professor of Surgery in the Philadelphia Polyclinic, Mütter Lecturer on Surgical Pathology of the College of Physicians of Philadelphia. With 39 illustrations. 162 pages. Price, $1.50. New York: D. Appleton & Co. 1899.

Dr. John B. Roberts, of Philadelphia, is a standard authority, and anything he writes attracts attention throughout the country. This small book on fractures embraces the author's experience and views after many years as a teacher and hospital surgeon.

Those who have been fortunate enough to attend a course in the Philadelphia Polyclinic have noticed with what precision and care Prof. Roberts adjusts a fracture. Many new ideas have been incorporated, and many old theories, inconsistent with present day surgery, have been omitted. A number of X ray cuts are employed to illustrate correct work. T. J. B.

